# On the Intentionality of Cultural Products: Representations of Black History As Psychological Affordances

**DOI:** 10.3389/fpsyg.2016.01166

**Published:** 2016-08-29

**Authors:** Phia S. Salter, Glenn Adams

**Affiliations:** ^1^Psychology and Africana Studies, Texas A&M UniversityCollege Station, TX, USA; ^2^Psychology, University of KansasLawrence, KS, USA

**Keywords:** Black History Month, collective memory, intentional worlds, culture, racial inequality

## Abstract

A cultural-psychological analysis emphasizes the intentionality of everyday worlds: the idea that material products not only bear psychological traces of culturally constituted beliefs and desires, but also subsequently afford and promote culturally consistent understandings and actions. We applied this conceptual framework of mutual constitution in a research project using quantitative and qualitative approaches to understand the dynamic resonance between sociocultural variance in Black History Month (BHM) representations and the reproduction of racial inequality in the U.S. In studies 1 and 2, we considered whether mainstream BHM artifacts reflect the preferences and understandings of White Americans (i.e., *psychological constitution of cultural worlds*). Consistent with the *psychological constitution* hypothesis, White American participants reported more positive affect, better recognition, and greater liking for BHM representations from the schools where White Americans were the majority than BHM representations from the schools where Black students and other students of color were the majority. Moreover, as an indication of the identity relevance of BHM representations, White identification was more positively associated with judgments of positive affect and preference in response to BHM representations from White schools than BHM representations from the schools where Black students were in the majority. In studies 3 and 4, we considered whether BHM representations from different settings differentially afford support or opposition to anti-racism policies (i.e., *cultural constitution of psychological experience*). In support of the *cultural constitution* hypothesis, BHM representations typical of schools where Black students were in the majority were more effective at promoting support for anti-racism policies compared to BHM representations typical of predominately White schools and a control condition. This effect was mediated by the effect of (different) BHM representations on perception of racism. Together, these studies suggest that representations of Black History constitute cultural affordances that, depending on their source, can promote (or impede) perception of racism and anti-racism efforts. This research contributes to an emerging body of work examining the bidirectional, psychological importance of cultural products. We discuss implications for theorizing collective manifestations of mind.

## Introduction

In 1926, Carter G. Woodson proposed a tradition of “Negro History Week” to commemorate and legitimize Black Americans' contributions to mainstream American society. The explicit motivation for this tradition was to shape not only pride within Black American communities, but also anti-racist consciousness throughout American society (Dagbovie, 2004). The tradition has subsequently become an increasingly mainstream event, especially after the United States Congress recognized February as Black History Month (BHM) in 1986. As mainstream American society has increasingly appropriated BHM, this cultural tool has evolved to serve a variety of different purposes (Pitre and Ray, 2002). Indeed, scholars debate whether current BHM commemorations serve Woodson's liberatory goals or are instead primarily a means for corporations to market goods to the Black community (e.g., posters from Budweiser with the slogan “this chapter of history brought to you by the king of beer”; Persinger, 2011; see also Franklin, 1997; Dagbovie, 2005). These mainstream appropriations make clear that commemoration of BHM is not simply about disinterested documentation of Black American histories. Instead, the variety of forms and purposes suggest that representations of BHM may function as *cultural affordances* (Kitayama and Markus, 1999; Kitayama et al., 2006; see Gibson, 1977): that is, cultural tools that make possible particular beliefs, motivations, and actions.

The present research applies a cultural psychology analysis to consider how displays for BHM reflect and promote identity-relevant action. Although the research involves a comparison of cultural products from Black American and White American spaces, what makes this a cultural psychology project is a focus on the mediation of human experience via cultural tools (Vygotsky, 1978; Wertsch, 2002; Rogoff, 2003).

## Cultural psychology: the study of intentional worlds

In his field-defining work, Shweder (1990) famously defined cultural psychology as the study of *mutual constitution*: how “psyche and culture …make each other up” (p. 1). Subsequent work in cultural psychology has tended to emphasize one direction of this mutual constitution dynamic: the *cultural constitution of psyche*. This direction emphasizes that species-typical patterns of mind are not just natural, but instead emerge as people engage with structures of mind-in-context that provide the necessary ecological scaffolding for human experience (Markus and Kitayama, 1991; Kim and Markus, 1999; Heine and Lehman, 2004; Adams et al., 2010). Subsequent work has tended to neglect the other direction of the mutual constitution dynamic: the *psychological constitution of cultural worlds*. This direction emphasizes that the everyday ecology of the human organism is likewise not just natural, but instead emerges through everyday actions as people realize (i.e., *make real*; Berger and Luckmann, 1966/1992; Moscovici, 1984) their understandings and desires (e.g., Kim and Markus, 1999; Markus et al., 2006; Adams et al., 2010).

The relative neglect of the psychological constitution process is striking given that, a few paragraphs later, Shweder (1990) defined cultural psychology as “the study of intentional worlds” (p. 3), that is, “human artifactual worlds populated with products of our own design” (p. 2). He proposed that “a sociocultural environment is an intentional world …as long as there exists a community of persons whose beliefs, desires, emotions, purposes, and other mental representations are *directed at it and are thereby influenced by it* (Shweder, 1990; p. 2; emphasis added). To refer to cultural worlds as *directed* (or constituted) is to say that everyday realities are not neutral; instead, people infuse them with the charge of their beliefs and desires. To refer to cultural worlds as *directive* (or constituting) is to say that everyday realities are not psychologically inert; instead, they afford some forms of experience, constrain other forms, and direct action toward particular ends.

### History narratives as directed cultural products

In this research, we apply ideas about the intentionality of everyday worlds to a particular class of cultural-psychological products: historical narratives. Historical narratives are not often firsthand experiences of seminal past events (Adamczyk, 2002); instead, they are dynamically constructed, collective memories. When sources present historical narratives as “just the facts” or “objective” accounts of the past, they obscure how reconstructions of the past must necessarily omit some details (Loewen, 1995; Trouillot, 1995).

Among the most important influences on production of *historical memory* are social identity processes. Social identification affects (re)production of historical narratives by influencing people to deny, minimize, silence, or otherwise de-select information about collective misdeeds or other unflattering events. People experience motivations to reconstruct the past in an identity-favorable light to avoid negative feelings and emotions associated with threats to collective identity (e.g., Branscombe and Miron, 2004; Wohl et al., 2006; Morton and Sonnenberg, 2011). These motivations can lead people who are high in collective identification (either as a stable individual difference or temporary situational affordance) to minimize the negative consequences of collective misdeeds (Doosje et al., 1998) or to use strategies of psychological distancing to minimize their relevance for present events (Pennebaker and Banasik, 1997; Kurtiş et al., 2010). Besides these relatively “hot” cognitions or motivated forms of minimization and denial, dominant constructions of social identity often have celebratory connotations that lead people to recall more positive (or fewer negative) features of the collective past, regardless of their individual motivations (Sahdra and Ross, 2007).

The influence of social identity on production of historical memory has implications for the psychological constitution of cultural reality. When social identity processes lead people to (re)produce some historical narratives rather than others, the result is not merely the endpoint of an action sequence. Instead, identity-relevant action exerts selective pressure on the reproduction of cultural reality. The resulting narratives carry the psychological charge of particular beliefs and desires (and not others), and this charge circulates with these narratives beyond the immediate time and place of their production. The important implication is that the presence and absence of particular history narratives in everyday cultural ecologies are not the product of happenstance (Baumeister and Hastings, 1997; Pennebaker and Gonzales, 2009); instead, prominent collective narratives bear the influence of identity-relevant concerns regarding what is true and desirable.

### History narratives as a directive cultural force

Representations of history provide the scaffolding for conceptions of nationhood and other collective identities (Reicher and Hopkins, 2001; Eyerman, 2004; Liu and Hilton, 2005; Hammack, 2010). They constitute tools that provide distinctiveness in social comparisons with other nations; confer legitimacy to norms, moral codes, and laws; and facilitate the assimilation (or rejection) of marginalized groups via official policies of monoculturalism or multiculturalism (Liu and Hilton, 2005). History representations provide access to a collective story around which individuals can locate their personal and collective selves. For example, the trauma of slavery is a collective memory that has served as a basis for African American identity (Eyerman, 2004). Engagement with representations associated with collective memory of American slavery—not the actual experience of slavery—influences how Americans of African descent see themselves in relation to other groups (e.g., collectively identifying as African American).

Beyond collective identity construction, history narratives also impact identity-relevant perception and action. Of particular relevance to the present work is research that shows how differences in knowledge about past racism can mediate divergent perceptions of racism in contemporary societies (Nelson et al., 2013). That is, persistent identity-relevant differences in perception of racism can reflect identity-relevant differences in historical knowledge. Other research demonstrates that the salience of historical narratives can impact support for reparative action. For example, low-identified Dutch participants advocated more aid to an Indonesian cause when aspects of Dutch history (including colonial occupation of Indonesia) were salient than when these aspects of history were not salient (Doosje et al., 1998). In summary, historical knowledge can be a directive force that impacts construction of current events and subsequent responses (e.g., reparative action, apologies, or compensation).

## Power and racial privilege in cultural production

An important implication of an intentional worlds analysis is that identity-relevant products prominent in a particular cultural setting are likely to reflect and promote identity concerns of dominant groups. Applied to the present study, historical narratives in mainstream American settings are likely to reflect and promote beliefs and desires of the dominant, White American majority. Moreover, the relationship between identity concerns and historical representation may be amplified in cases, like institutionally sanctioned commemoration of BHM, where the events under consideration—enslavement, systematic rape and torture, terroristic violence, apartheid/segregation, and other crimes against humanity—disrupt celebratory or glorifying narratives of nation and are therefore potentially threatening to dominant-group constructions of identity. Given social pressures to commemorate events associated with BHM, the impact of dominant-group identity concerns on cultural reproduction may lead to the evolution of relatively “sanitized” representations of history that silence or minimize discussions of oppression (Brown and Brown, 2010; Kurtiş et al., 2010). One strategy for production of sanitized representations of history is to frame discussions about racism in terms of multicultural tolerance, diversity, or other ways consistent with the “prejudice problematic” (e.g., Wetherell, 2011) rather than focus on the ongoing legacy of systemic expropriation, exploitation, and violent oppression (Adams et al., 2008; Wright and Lubensky, 2009; Hammack, 2011). Another strategy for production of sanitized representations of history is to highlight individual achievement (despite un-named barriers) rather than collective struggle (to eliminate barriers; Banks, 1999; Pitre and Ray, 2002). Besides deflection of attention away from structural barriers, the emphasis on heroic individual achievement has the added advantage of resonating with dominant group ideologies—specifically, colorblindness, meritocracy, protestant work ethic—that reinforce the atomistic construction of racism in terms of the prejudice problematic (e.g., Levin et al., 1998; Jost et al., 2004). To the extent that mainstream historical narratives promote denial of racism in contemporary society by omitting narratives of oppression and other objectionable events (e.g., genocide, slavery; Loewen, 1995; Schick and St. Denis, 2005), they constitute intentional worlds of racism that further perpetuate racial domination.

## Overview of the present research

We conducted four studies that explore how representations of BHM function as cultural-psychological tools for identity-relevant perception and action. The design of the research relies heavily on the idea of cultural realities as psychologically constituted, intentional worlds that both reflect and promote identity-relevant, community-specific beliefs and desires. This design emphasizes two ideas regarding the “intentional” character of Black History representations. The first idea is that representations of Black History are not “natural” or “objective” accounts of the past. Instead, these cultural products bear the psychological imprint of community-specific beliefs and desires. We refer to this idea as the *psychological constitution* hypothesis. The second idea is that representations of Black History are not neutral or inert. Instead, these representations carry a charge that affords identity-relevant action in the service of community-specific goals. We refer to this idea as the *cultural constitution* hypothesis. The hypotheses are complementary and reflect the bi-directional, mutually-constituted character of psyche and culture.

### Psychological constitution of cultural worlds

To investigate the psychological constitution hypothesis, we first conducted a field study (Study 1) in which we compared commemoration of BHM in effectively segregated high schools: one set of “White-Majority” schools where European American students were a near-exclusive majority, and another set of “Black-Majority” schools where African American and Latina/o students (i.e., racial/ethnic minorities across the U.S. in general) were in the numerical majority^1^. Consistent with discovery phases of the scientific method, the goal was to explore the everyday material affordances for BHM commemoration in different school communities.

In Study 2, we exposed White American university students to photographs of BHM displays that we documented in Study 1. An *intentional worlds* framework suggests that BHM representations from different communities are material expressions of different understandings and desires: that is, identity-consistent traces of beliefs about what is right and good deposited in the structure of everyday ecologies. To the extent that cultural products in White American settings carry understandings that resonate with White Americans beliefs and desires, the psychological constitution hypothesis proposes that (1) White American undergraduates will express greater familiarity and preference for BHM displays from White-Majority schools than Black-Majority schools and (2) this pattern of differential recognition and preference will vary as a function of collective identification.

### Cultural constitution of psychological experience

To investigate the cultural constitution hypothesis, we conducted a pair of between-participants experiments in which we exposed White American undergraduates to one of two treatment conditions or a third, control condition. In the treatment conditions, participants either engaged with photographs of BHM displays (Study 3) or facts reflecting BHM themes (Study 4) that we observed in either White-Majority schools or Black-Majority schools. We then examined the consequences of exposure (or not) to these treatments for two outcome variables related to Woodson's original motivations for proposing Negro History Week: (1) perceptions of racism in US society and (2) support for action on behalf of racial justice. Resonating with the idea that preferred representations systematically promote “preferred” identity-relevant outcomes, the cultural constitution hypothesis proposes that BHM displays or themes from Black-Majority schools will be more effective than BHM displays or themes from White-Majority schools (and control conditions) at increasing perception of racism and support for remediation of racial injustice.

Implicit in our theoretical framework is the idea that BHM representations from Black-Majority schools afford support for anti-racism policies because they alert viewers to the ongoing significance of racism in contemporary U.S. society. This implies a mediation hypothesis whereby the effect of BHM representations on perception of racism exerts an indirect, facilitative effect of BHM representations on support for remedial social justice policy. The design of Studies 3 and 4 permits a test of this hypothesis.

## Study 1

Study 1 was a field study in which we used qualitative methods to investigate the ways in which local high schools commemorated BHM. [The first author] visited research sites to interview school personnel, to conduct naturalistic observations of material artifacts and display practices, and to collect photographic documentation of BHM displays. The epistemological goal was to sketch the cultural ecology of BHM commemoration in keeping with initial phases of the scientific method that emphasize discovery and naturalistic description of everyday worlds (see Barker, 1968). An institutional review board evaluated and approved all studies.

## Methods

[The first author] contacted 16 schools in a large Midwestern U.S. metropolitan area and requested permission to observe educational displays during the month of February. Of these 16 schools, 12 had centralized BHM displays. In 7 of these 12 high schools, White students were the majority (*M* = 86%; range = 84–92%); in the remaining 5 schools, White students were in the minority (*M* = 16%; range = 2–28%). [The first author] documented “official,” centralized displays located in libraries, cafeterias, classrooms, and hallways, but also photographed any posters, pictures, signs, or miscellaneous items (e.g., televised daily announcements) related to BHM commemoration throughout the schools.

Current conventions for writing and publication in psychological science require that authors provide information on the identity background of participants; however, current conventions do not (yet) require the disclosure of author or researcher identity. Indeed, conventional practice implies that author or researcher identity is unremarkable in “normal” circumstances and only becomes worthy of remark in “abnormal” circumstances where either participants or researcher identity deviates from the putative White standard. In contrast to this racialized imagination of the research process, a cultural-psychology perspective prescribes greater attention to researcher identity and its impact on the resulting science (e.g., Adams and Stocks, 2008). Although applicable to all psychological science, this prescription of reflexive disclosure is especially relevant in field observations, interviews, or other research that requires extensive interaction between researcher and participant (Blauner and Wellman, 1998; Wertz et al., 2011; also see Collins, 2002 for discussion of positionality). In keeping with this prescription, we note that all field research visits and empirical observations that we report in Study 1 were the work of the first author, an American Black woman whose own educational background included experiences in a predominantly Black high school (2 years), a predominately White high school (2 years), a predominately White college (3.5 years), and study for a brief period at a historically Black college (1 semester). The second author contributed to the design, analysis, and reporting of the present research. He is a White American man whose educational background included experience in predominantly White institutions and extensive work (total of more than 6 years) in African and other “Majority-World” settings of the Global South.

## Results and discussion

This field study yielded several noteworthy observations about BHM commemoration practices. Brief interviews with staff (*N* = 17) revealed that responsibility for creating BHM displays often fell to school librarians, but creators also included students, teachers, guidance or diversity counselors, and (in one case) “the only Black staff” person. Displays generally emphasized Black icons from the past (e.g., Martin Luther King, Jr.), but some also included contemporary figures (e.g., Oprah Winfrey and Bill Cosby). The most frequent color scheme was a pan-African motif (i.e., red, black, green, and gold), but Kente-cloth motifs and multicultural rainbow were also common. Besides these observations, four major themes emerged.

### Theme #1: commercialized commemoration

**Figure 1:**
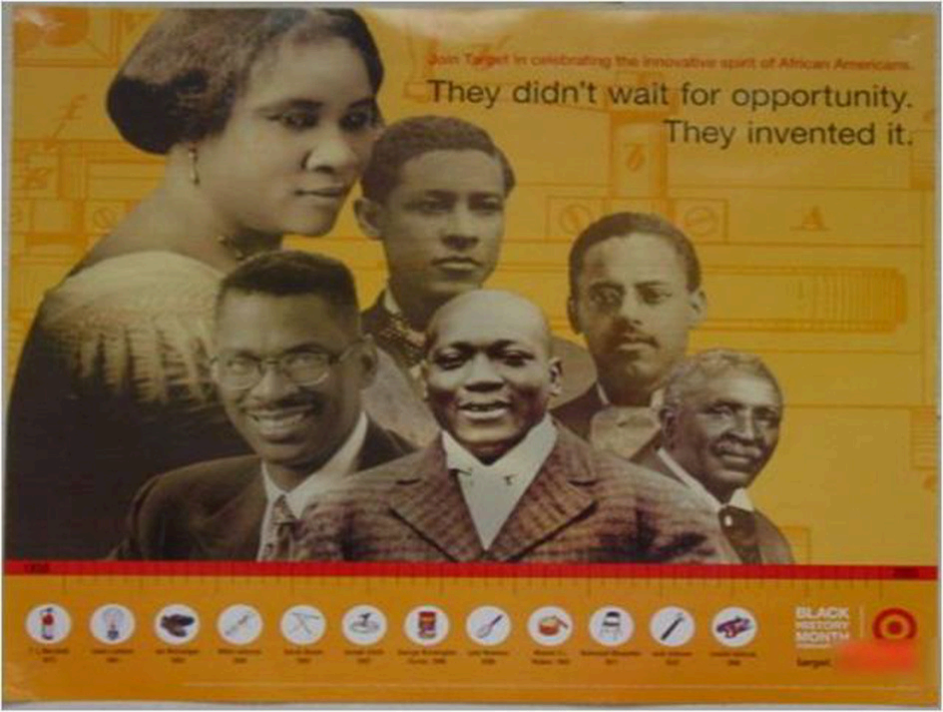
**Example of a commercially-produced commemoration of Black History Month Representation by Target, Inc., Study 1**.

One notable theme was the frequency of commercially available, “pre-packaged” BHM materials. A particularly noteworthy example of such materials is a poster from the department store chain, Target (see Figure 1), which [the first author] observed in 25% (i.e., 3 of 12) of the schools that she visited. The poster shows images of African American inventors and products that they invented. Accompanying the images are two textual statements. One statement in small print invites viewers to “Join Target in celebrating the innovative spirit of African Americans.” The other statement in large print adds, “They didn't wait for opportunity. They invented it.”

**Figure 2:**
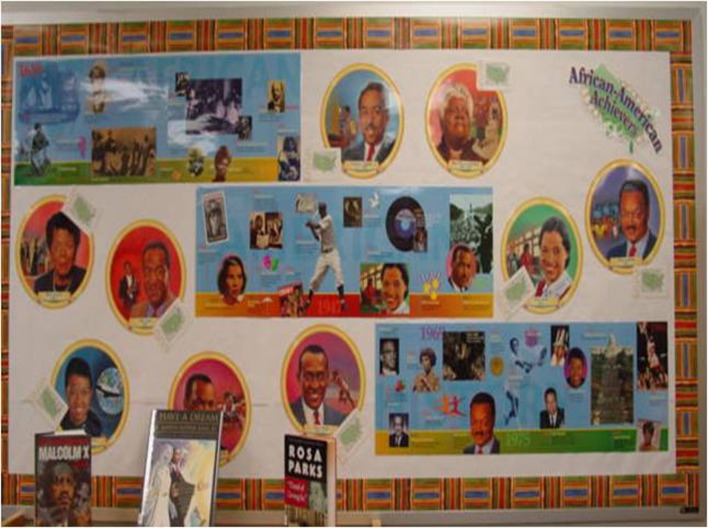
**Example of a commercially-produced centralized display for Black History Month from a White-Majority school, Study 1**.

The Target poster is typical of pre-packaged materials and illustrates well the celebratory emphasis on heroic achievements of educators, inventors, athletes, and entertainers, with corresponding silence about contextual circumstances or structural barriers that required heroic action. Pre-packaged materials sometimes had information about each actor's contributions, but often included only the person's name and picture. With the exception of one display that included a timeline, the kits exclusively showcased people rather than places or events (for example, see Figure 2).

### Theme #2: celebrating diversity

**Figure 3:**
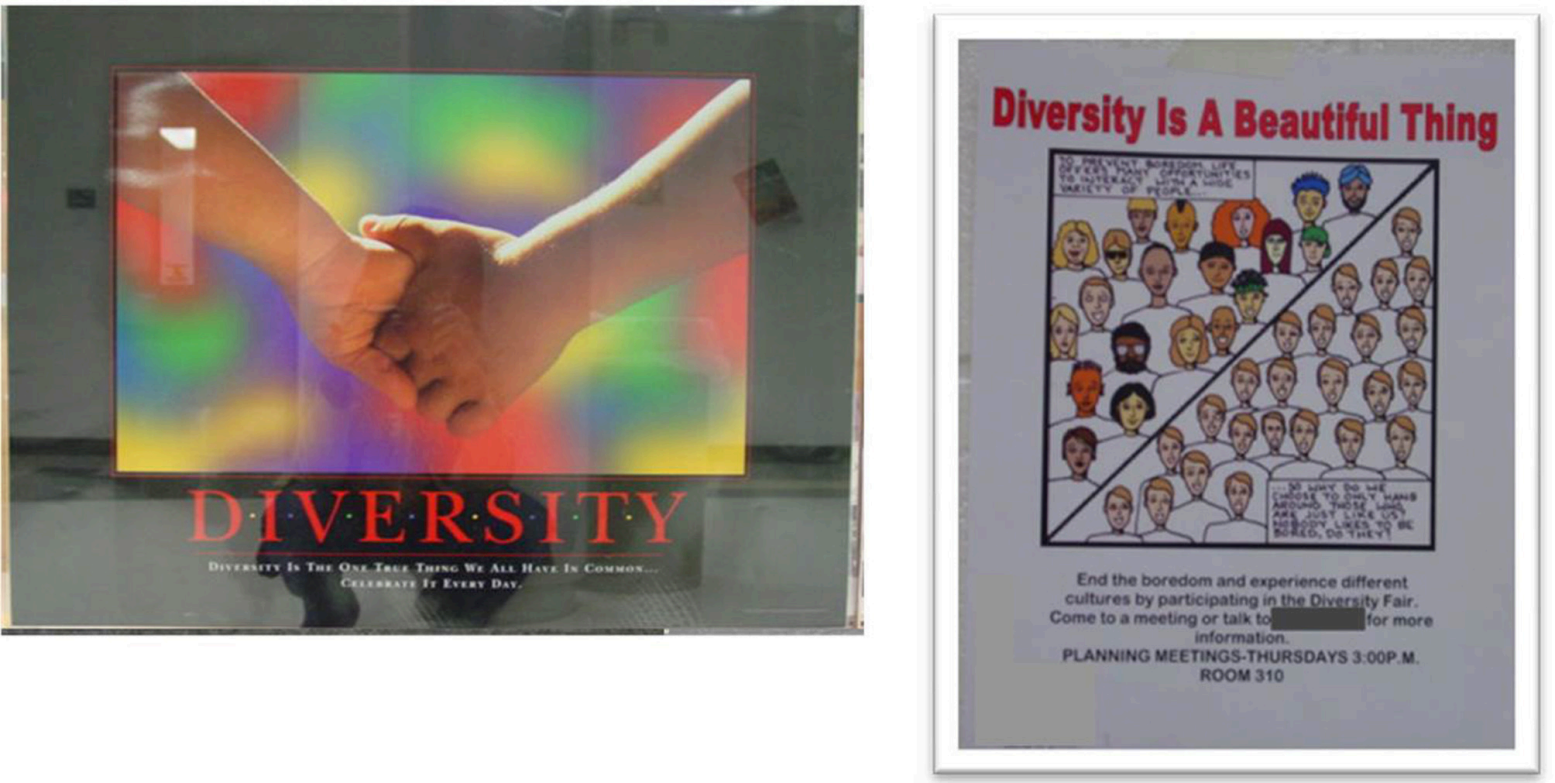
**Examples of materials from a White-Majority school that linked Black History Month to larger issues of cultural diversity, Study 1**.

Materials at some schools linked BHM to larger issues of cultural diversity rather than “Black history” specifically. Examples of this theme include the posters in Figure 3, which not only make explicit reference to diversity (e.g., “Diversity is the one true thing we all have in common”), but also provide a visual representation of the theme through use of the multicultural rainbow motif or depiction of human faces. At one school where White students were the majority, the librarian explained that the diversity theme purposefully connected students to the display: “We tried to pull books to reflect hopefully every student in the building. We had books in there as far as different races go, different religions …we have multiracial, gay and straight, any kind of student.” Implicit in such statements is a concern that the purpose of BHM commemoration is not self-evident in schools without a large Black student population, and creators of displays may emphasize broader themes of diversity rather than history to make the occasion relevant to all students.

### Theme #3: celebrating achievements

Another notable theme was the extent to which BHM representations focused on the individual achievements of African Americans. In many cases, materials highlighted individuals who were famous “firsts” (e.g., Jackie Robinson, famous for breaking the racial barrier within American baseball). Such representations alluded to racial barriers without specifically referring to them. There was also an emphasis on Civil Rights activists (i.e., Martin Luther King, Jr.; Rosa Parks); though, the racist conditions precipitating the Civil Rights Movement minimally figured into commemoration materials. Interview respondents were sometimes explicit about this de-emphasis of racism themes. One Diversity counselor (and designer of a BHM display at a majority White school) expressed agreement with a co-worker who had described her ideal BHM display as one that focused on positive contributions:

Our students learn so much about slavery and different ways that people have been oppressed, but for BHM, in particular, to me that's a time to celebrate. Rather than watching the 12 h of Roots and be sad and defeated and cry—which is important, but not necessarily when it comes time to celebrate—like I'd rather, if the kids are going to watch things, for instance, I'd like them to see *Something the Lord Made* with Mos Def …or *Soul Food*. Movies that really the students can connect to and maybe learn something about themselves or the kids that they have class with.

From this perspective, BHM provides teachers with an opportunity to celebrate African American culture. However, efforts to go beyond themes of racism and oppression in America's history may reinforce somewhat stereotypical portrayals of contemporary African American communities. This may especially be the case when individuals are presented as the exception to the rule.

### Theme #4: acknowledging racism

**Figure 4:**
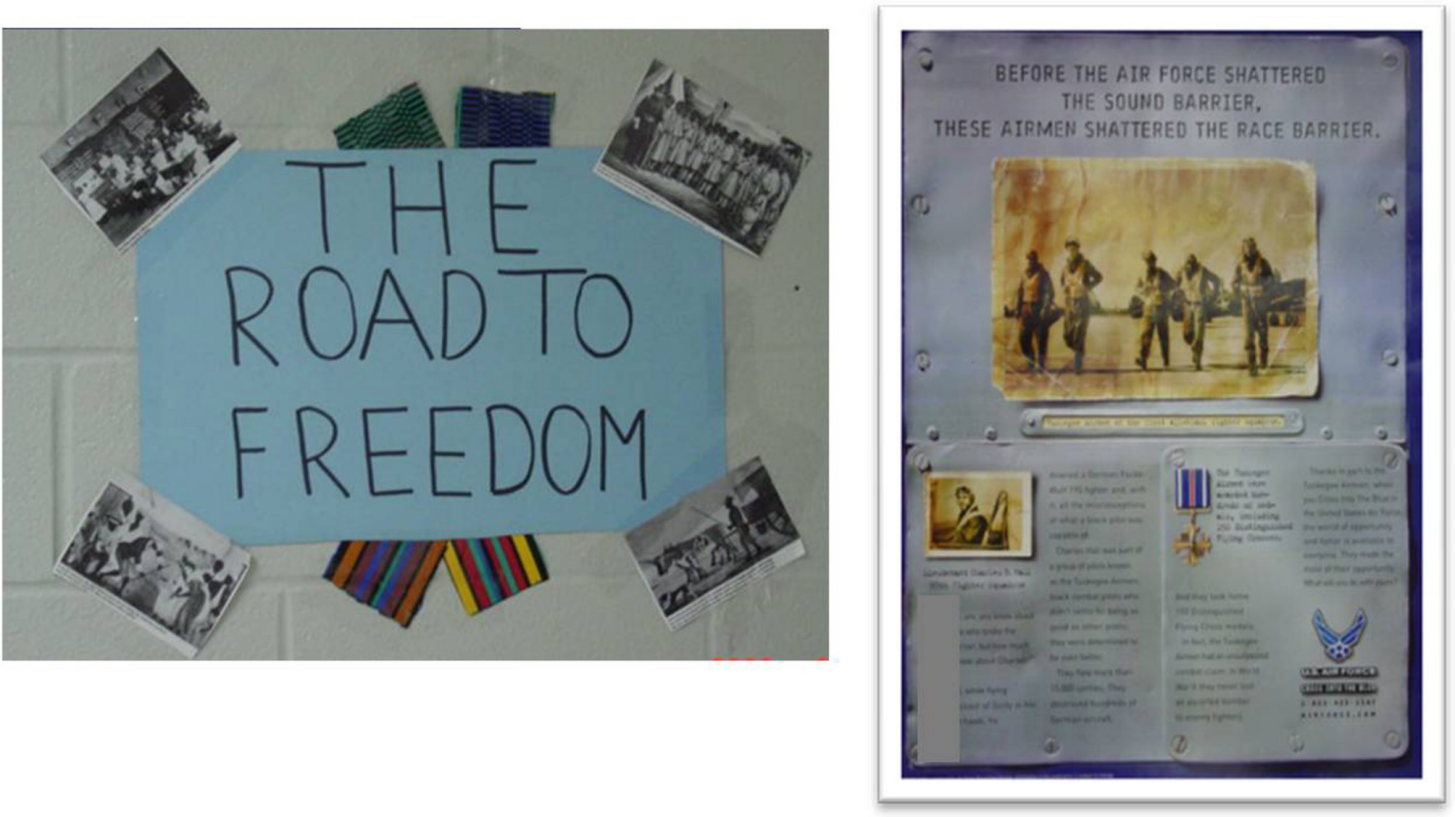
**Examples of materials from a Black-Majority school that linked Black History Month to historical racism, Study 1**.

The theme of racism was typically absent from BHM displays aside from serving as background for celebrating achievements. Despite this general de-emphasis of racism, some schools did have additional representations around the school that made more explicit reference to racism in discussions of slavery, “Jim Crow” segregation, and events surrounding the Civil Rights Movement (e.g., freedom rides; see Figure 4). Furthermore, interview responses clarified that presentation of racism-relevant content was not always in the form of visual displays, and some interviewees talked about integrating “the struggle” in Black History into their lessons during the month. A librarian, who was also teaching reading enhancement at a predominately Black school, indicated that lessons are sometimes timed so that they occur during BHM:

I always try to make things …sort of relevant to the kids—and so the next unit we're going to do is on New Orleans. And, of course [Hurricane] Katrina and all of that stuff that happened there that affected African Americans on a greater scale than it did other groups of people and so that's another way of bringing Black history—that is current—that kids can relate to.

Within this conception, BHM is the time to discuss various manifestations of racial oppression in contemporary events that differentially impacted Black communities (i.e., Hurricane Katrina).

### Variation in themes across research settings

Although there were many commonalities across the schools, a primary interest in the present research is the possibility of systematic differences in representations of BHM as a function of the community the school serves. A precise quantification of variation in themes was impractical for two reasons. First, the multiplicity of different and sometimes conflicting representations within a given school made it difficult to quantify precisely the extent to which a school's commemoration of BHM expressed relevant themes. Second, the small sample size precluded the use of statistical tests for differences as a function of community setting. Even so, the totality of qualitative evidence from interview responses, naturalistic observation, and photographic documentation affords more holistic conclusions about the distribution of themes as a function of community settings. A summary of these ethnographic observations appears in Table 1. Taken as a whole, this evidence suggests that tendencies to emphasize collective self-enhancement over collective self-knowledge—that is to focus on values of diversity vs. history and to prioritize individual achievements over historical barriers—were all stronger in White-Majority schools than in Black-Majority schools.

**Table 1:** **Black History Month themes in central displays and other materials by school site**.

**Site**	**Population (%)**		**Centralized BHM display**		**Themes**			
	**White**	**Black**	**Title**	**Location**	**COM**	**DIV**	**ACH**	**RAC**
1	92	3	“Celebrate Diversity and our Common Heritage”	Library book display		✓		
2	91	2	“Black History Month: celebrating a Rich Heritage”	Hallway bulletin board		✓	✓	
3	92	3	None		✓	✓	✓	
4	90	1	“African American Achievers”	Library bulletin board	✓		✓	✓
5	87	4	None			✓	✓	
6	88	5	None					✓
7	85	9	No title/slogan; featured historical figures	Hallway bulletin board	✓		✓	
8	87	6	None					
9	84	7	No title/slogan; featured historical figures	Library book display	✓		✓	
10	84	6	“The price of freedom is visible here”	Library book display				
11	75	8	“Celebrate Black History Month”	Classroom bulletin board	✓		✓	
12	39	43	No title/slogan; featured writers	Library book display			✓	
13	29	65+	“Black History Month”	Hallway display case			✓	
14	27	29^*^	“African American history”	Hallway bulletin board	✓		✓	✓
			No title/slogan; featured historical/present-day figures	Cafeteria display case			✓	
15	15	76	None		✓		✓	✓
16	10	60	“Remembering Rosa Parks”	Hallway bulletin board:			✓	✓
17	2	96	“Black Inventors and their Inventions”	Hallway bulletin board	✓		✓	✓

*BHM, Black History Month. COM, Commercialized Commemoration; DIV, Celebrating Diversity; ACH, Celebrating Achievements; RAC, Acknowledging Racism. +Site 13 was added after initial data collection for Study 1, but was considered in Study 1 analyses and utilized in Study 3*.

* *Site 14 is the school in which Latin@/Hispanic students were in the majority (40%)*.

Although necessarily tentative, these conclusions are consistent with education research on history representations in the U.S. (Banks, 1999; Alridge, 2006; Journell, 2008). For example, an analysis of social studies standards in nine states revealed two primary themes—“Inclusion of Individuals” and “Oppression and Emancipation vs. Culture and Contribution”—(Journell, 2008) that resonate with observations of the present study. Similarly, a comparison of historical knowledge revealed differences as a function of school setting such that White students were less likely than Black students to cite the Civil Rights Movement, Civil War, and slavery as important historical events (Epstein, 2000).

The objective of Study 1 was to provide a qualitative sense for everyday practices of BHM commemoration in different school communities (Marecek et al., 1997; Denzin and Lincoln, 2012). Although this objective is especially appropriate for the naturalistic observation and description stages of the scientific method, the exploratory character of this initial study precluded strong conclusions about the distribution of themes in BHM displays. We return to this issue with respect to the present materials in Study 3, but more definitive conclusions about the distribution of themes require additional analysis of materials from a larger sample of schools. This remains a direction for future research.

## Study 2

Study 2 considers the psychological constitution hypothesis. To investigate this idea, we exposed White American undergraduates to photographs of BHM displays from both White-Majority and Black-Majority schools. In response to each photograph, we asked participants to make ratings of recognition and liking without providing information about the source of each display (i.e., whether from a White-Majority or Black-Majority school). If cultural products from White-Majority schools resonate better with White American beliefs and desires than do cultural products from Black-Majority schools, then White American participants will express stronger recognition and liking of BHM displays from White-Majority schools than BHM displays from Black-Majority schools. Moreover, this pattern of responses will bear traces of relevant social identity concerns. Specifically, White American identity will be more positively (or less negatively) related to positive affect, liking, and recognition for ratings of White-Majority displays than for ratings of Black-Majority displays.

## Method

### Participants

A total of 52 undergraduates from a predominately-White, Midwestern American university ranged in age from 18 to 30 years of age (*M* = 19.22, *SD* = 1.98). Of participants who indicated race or ethnicity, fewer than 10% indicated a non-White race or ethnicity (Asian/Asian American, 2; African American/Black, 2; Hispanic/Latino, 1). We report analyses using only data from the 47 participants (27 men and 18 women) who indicated White and American identities.

### Procedure

We recruited participants from an introductory psychology subject pool. A White research assistant administered the study. Participants viewed a PowerPoint presentation containing photographs of the centralized BHM displays (for example, see Figure 4) from the seven high schools with majority White Student populations (i.e., White-Majority condition) and the five high schools where American students of color were in the majority (Black-Majority condition). We assigned participants at random to view these displays in one of two, alternating-order conditions (i.e., with either a White-Majority or Black-Majority display presented first). Participants completed ratings of affective responses, familiarity, and liking as they viewed each display. Participants were unaware that the displays came from schools with different racial/ethnic profiles. After completing the rating task, participants completed measures of identification and demographics.

### Materials

#### Affective responses

We measured affective responses by adapting items from the Positive and Negative Affect Scale (PANAS; Watson et al., 1988). Immediately after viewing each display (and before they viewed the next display), participants rated six positive emotions (e.g., *proud*; Cronbach's α = 0.96 and 0.95 for White-Majority and Black-Majority displays, respectively) and six negative emotions (e.g., *guilty*; Cronbach's α = 0.91 and 0.95 for White-Majority and Black-Majority displays, respectively) on a scale from 1 (*not at all*) to 5 (*extremely*) in response to the following prompt: “Indicate to what extent you feel this way, right now, that is, at the present moment.”

#### Display ratings

Participants responded to six evaluative questions using a 7-point scale ranging from 1(*not at all*) to 7 (*very much*). A principal components analysis using varimax rotation yielded two reliable factors. A first, “liking” factor consisted of 4 items—e.g., *How much do you like this display* and *To what extent would you like to see this display in your former high school*—that accounted for 73.15% of the variance (Cronbach's α = 0.89 and 0.90 for White-Majority and Black-Majority displays, respectively). A second, “recognition” factor consisted of 2 items—*How familiar are you with the contents of this display* and *To what extent does the overall display present the material accurately—*that accounted for 12.39% of the variance (Cronbach's α = 0.80 and 0.77 for White-Majority and Black-Majority displays, respectively).

#### Identification

We adapted the 4-item *identity* subscale of the Collective Self Esteem Scale (CSE; Luhtanen and Crocker, 1992) to assess racial/ethnic identity (Cronbach's α = 0.84)^2^. This subscale measures individual differences in importance of a social identity category for self-definition (e.g., *The racial/ethnic group I belong to is an important reflection of who I am*). Participants responded to these questions with a 7-point Likert scale ranging from 1 (*not at all*) to 7 (*very much*).

## Results and discussion

To investigate whether representations of BHM from different school settings carry community-consistent understandings and preferences, we conducted within-subjects analyses of ratings about positive and negative emotions, liking, and recognition by White American participants in response to White-Majority and Black-Majority displays. To explore the identity-relevance hypothesis, we tested whether the relationship between identification and responses to displays differed as a function of community source.

### Affective responses

We conducted a 2 (Display Source: White-Majority school, Black-Majority school) × 2 (Affect Valence: Positive, Negative) repeated measures ANOVA to investigate the effect of display source on affective responses. Results revealed main effects of display source, *F*_(1, 42)_ = 19.88, *p* < 0.001, $\eta_{\text{p}}^{2}=0.32$, and valence, *F*_(1, 42)_ = 105.33, *p* < 0.001, $\eta_{\text{p}}^{2}$ = 0.72, qualified by a Display × Valence interaction, *F*_(1, 42)_ = 21.33, *p* < 0.001, $\eta_{\text{p}}^{2}$ = 0.35. Follow-up analyses indicated a hypothesized effect of display source that was primarily true for positive emotion items. Participants reported more positive emotions while viewing White-Majority displays (*M* = 2.41, *SD* = 0.67) than while viewing Black-Majority displays (*M* = 2.05, *SD* = 0.67), *t*_(43)_ = 5.035, *p* < 0.001, *d* = 0.83. In contrast, participants indicated little negative emotion while viewing either Black-Majority displays (*M* = 1.20, *SD* = 0.36) or White-Majority displays (*M* = 1.13, *SD* = 0.21), *t*_(44)_ = −1.72, *p* = 0.092, *d* = 0.39. To summarize, something about viewing representations of Black History from White-Majority schools prompted White American undergraduates to feel better than they did after viewing representations of Black History from Black-Majority schools (i.e., where American students of color were in the majority)^3^.

### Display ratings

We conducted a 2 (Display Source: White-Majority school, Black-Majority school) × 2 (Evaluation Type: Liking, Recognition) repeated measures ANOVA to investigate the effect of display source on evaluations of liking and recognition. Results revealed the hypothesized main effect of display source, *F*_(1, 46)_ = 76.71, *p* < 0.001, $\eta_{\text{p}}^{2}$ = 0.63, and a main effect of evaluation type, *F*_(1, 46)_ = 38.57, *p* < 0.001, $\eta_{\text{p}}^{2}$ = 0.46, qualified by a significant Display × Evaluation Type interaction, *F*_(1, 46)_ = 49.31, *p* < 0.001, $\eta_{\text{p}}^{2}$ = 0.52. Although the difference between ratings of White-Majority and Black-Majority displays was large in both cases, paired-samples *t*-tests indicated that it was greater on the liking dimension (respective *M*s = 3.83 and 2.88, *SD*s = 0.77 and 0.82), *t*_(46)_ = 10.25, *p* < 0.001, *d* = 1.68 than on the recognition dimension (respective *M*s = 4.12 and 3.72, *SD*s = 0.86 and 0.86), *t*_(46)_ = 4.98, *p* < 0.001, *d* = 0.78. Consistent with the psychological constitution hypothesis, these White American participants expressed relatively high recognition and liking of BHM material from White-Majority schools, but they expressed less recognition and profoundly less liking of BHM material from Black-Majority schools.

### Identity relevance

What accounts for the pattern of observed differences in affect, liking, and recognition as a function of display source? Although there might be many factors that differ systematically across sets of BHM displays, an intentional worlds analysis suggests that ratings diverge, in part, because the displays resonate differently with beliefs and desires associated with White American identity concerns. To evaluate this feature of the psychological constitution hypothesis, we conducted one-tailed tests of within-subject differences in correlation coefficients.

**Figure 5:**
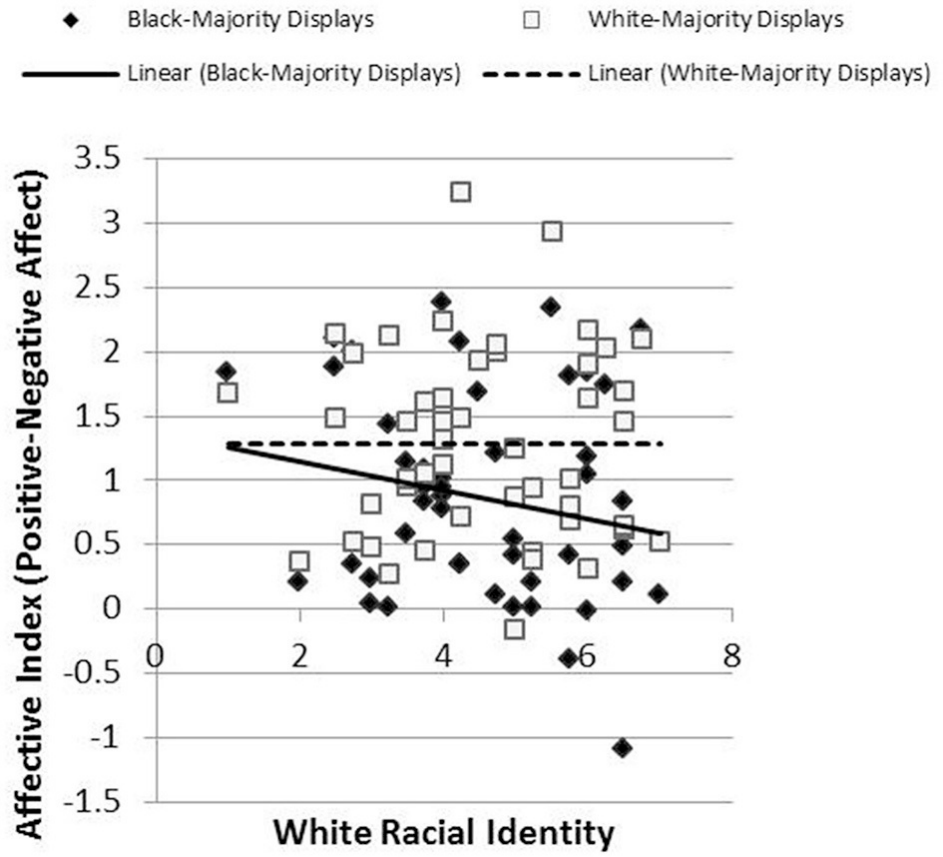
**Relationship between racial identity and affective responses to Black-Majority and White-Majority Black History displays, Study 2**.

**Figure 6:**
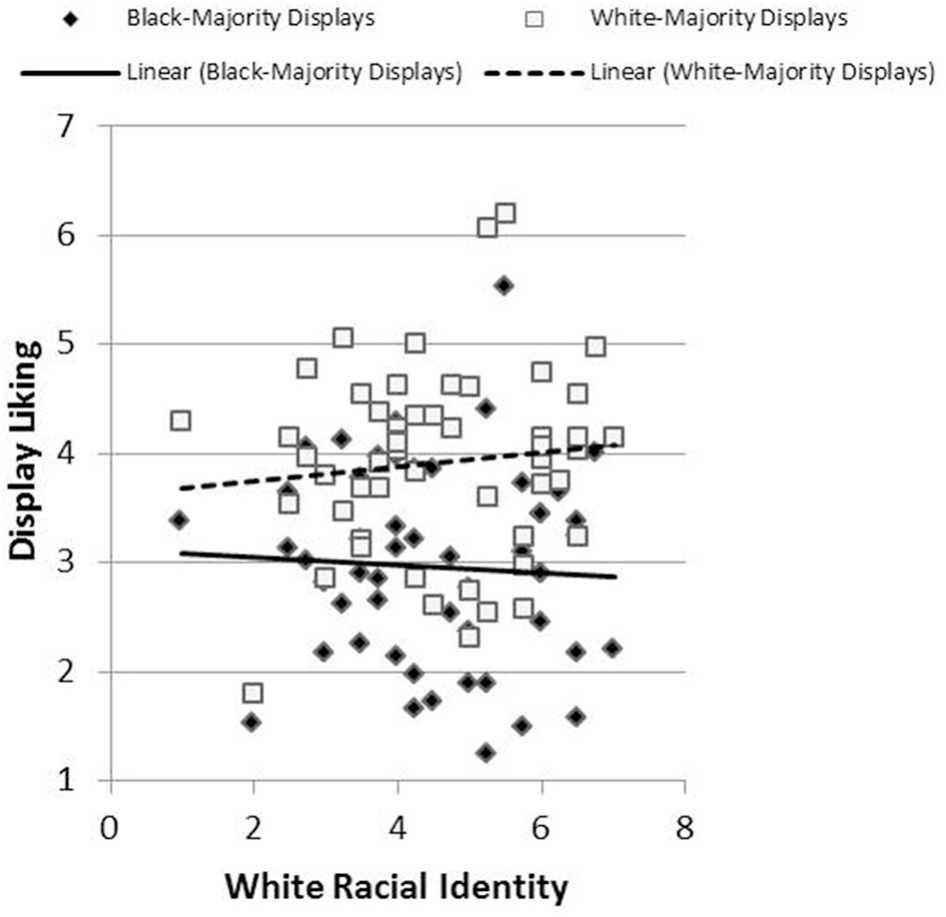
**Relationship between racial identity and display liking among Black-Majority and White-Majority Black History displays, Study 2**.

**Figure 7:**
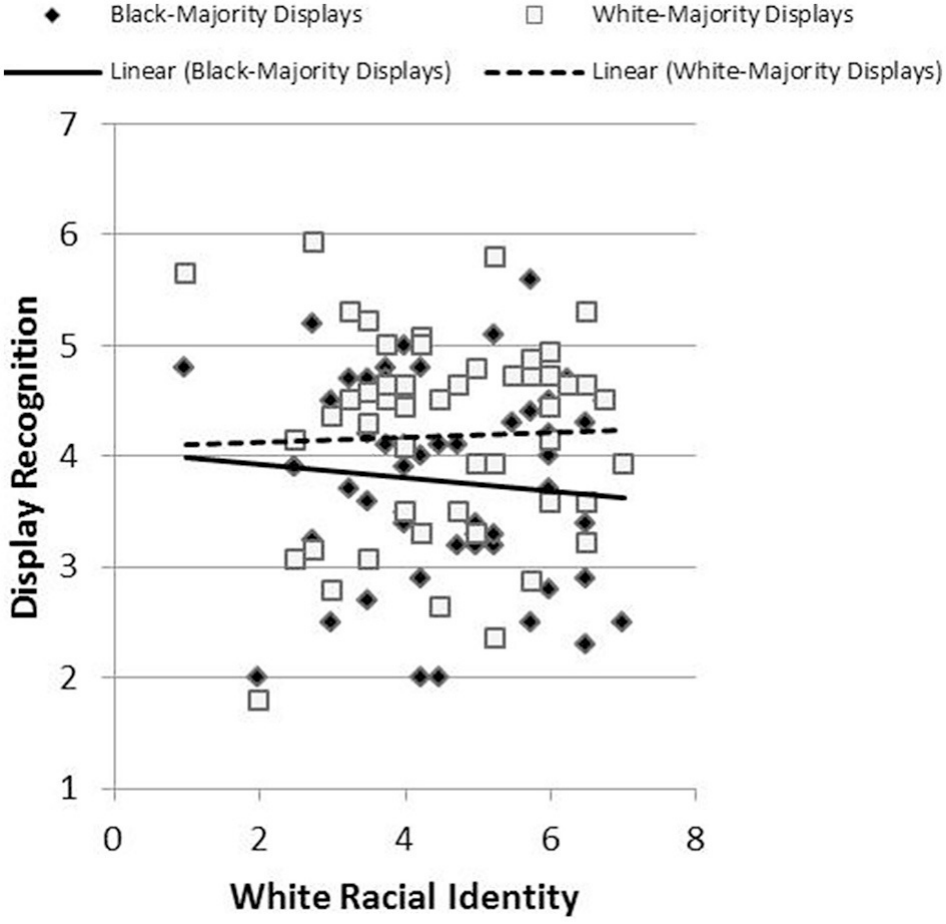
**Relationship between racial identity and display recognition among Black-Majority and White-Majority Black History displays, Study 2**.

Results revealed the hypothesized pattern. We created a single index of affective response for each participant by subtracting negative affect scores from positive affect scores. The relationship between racial identification and this affective index was less positive (more precisely, more negative) in reaction to Black-Majority displays (*r* = −0.273, *p* = 0.03) than in response to White-Majority displays (*r* = −0.057, *p* = 0.36), *t*_(43)_ = 1.76, *p* = 0.04, Cohen's *q* = 0.22 (Figure 5). Likewise, the relationship between racial identification and judgments of liking was more negative in reaction to Black-Majority displays (*r* = −0.134, *p* = 0.19) than in reaction to White-Majority displays (*r* = 0.089, *p* = 0.28), *t*_(44)_ = 1.92, *p* = 0.03, Cohen's *q* = 0.22 (Figure 6). Finally, the relationship between racial identification and judgments of recognition was more negative in reaction to Black-Majority displays (*r* = −0.068, *p* = 0.33) than in reaction to White-Majority displays (*r* = 0.083, *p* = 0.29), *t*_(44)_ = 1.61, *p* = 0.06, Cohen's *q* = 0.15 (Figure 7).

### Summary

Study 2 provides evidence of hypothesized preferences among White American college students for culturally consistent constructions of BHM. Although they were unaware of the source of displays, White American undergraduates exposed to photographs of BHM displays reported more positive affect, greater recognition, and greater liking for displays from White-Majority schools than displays from Black-Majority schools. An *intentional worlds* perspective suggests that these divergent reactions reflect the different affordances for identity present in displays from different settings. That is, displays from White-Majority schools are likely to rehearse content that affords White American students positive social identity, but displays from Black-Majority schools are likely to include themes that challenge White American identity. Although the exact features of BHM displays that afford divergent reactions remains an open question, evidence for the identity relevance of these features comes from hypothesized differences in the relationship between display ratings and White American identification. The relationship between White American identification and responses to Black History representations was significantly more negative in reaction to displays from Black-Majority schools than White-Majority schools.

## Study 3

Studies 1 and 2 provide evidence for one half of the intentional worlds framework, corresponding to the psychological constitution hypothesis. Specifically, results suggest that BHM representations in different communities are not neutral constructions of the past, but instead are directed products that realize and objectify community-specific beliefs and desires. Study 3 examines the other half of the intentional worlds framework, corresponding to the cultural constitution hypothesis. This idea proposes that realizations of BHM in different communities are not inert matter, but instead exert directive force that influences subsequent activity toward “desirable” or identity-serving ends.

## Method

### Participants

A total of 136 undergraduates (74 women and 62 men) from a predominately-White, Midwestern U.S. university ranged in age from 18 to 27 (*M* = 19.15, *SD* = 1.59). We retained data from the 123 participants who identified as American. Of these participants, 73.5% indicated White or European race or ethnicity (*n* = 100), 21% indicated another race or ethnicity (Asian/Asian American, 10; African/African American, 7; Hispanic/Latino, 4; American Indian, 1), and 5.5% did not indicate a category.

### Procedure

After obtaining informed consent, we randomly assigned participants to one of two BHM conditions (White-Majority or Black-Majority) or a third, control condition. In the Black-Majority condition, participants viewed photographs of six displays from Black-Majority schools^4^. In the White-Majority condition, we selected photographs of six displays at random from the original set of seven displays from White-Majority schools that participants rated in Study 2. To encourage engagement with displays, participants rated each display on dimensions of liking and recognition (as in Study 2). After engaging with the displays, participants completed a questionnaire. In the Control condition, participants completed the primary dependent measures (i.e., perceptions of racism, support for anti-racism policies) before viewing BHM photographs. Near the end of the survey, all participants responded to an open-ended prompt: *Please think about the History display that you liked the most. Please describe it and tell us what you liked about the display*. Last, participants completed the demographic questions.

### Dependent measures

#### Perceptions of racism

Four items (α = 0.73) assessed perception of racism in American society (adapted from Adams et al., 2006). Participants used a 7-point Likert scale (1, *not at all due to racism*; 7, *certainly due to racism*) to indicate the extent to which features of U.S. society were due to racism (e.g., *the disproportionate number of African Americans and Latinos in the criminal justice system*).

#### Support for anti-racism policies

Four items (α = 0.63) assessed endorsement of policies aimed to address racial inequalities in the U.S. (e.g., *Public and private universities should make every effort to attract qualified minority students*). Participants indicated the extent to which they agreed with the policies on a 7-point Likert scale (1, *Strongly disagree*; 7, *Strongly agree*).

## Results and discussion

To provide focused tests of the hypothesized effect of the historical representation manipulation on outcomes of interest, we conducted orthogonal planned contrasts with codes of (−1/3, −1/3, 2/3,) and (−1/2, 1/2, 0) for control, White-Majority, and Black-Majority representation conditions, respectively. The first contrast tested the primary hypothesis that Black-Majority representations will produce greater perceptions of racism in ambiguous events and endorsement of anti-racism policy than the control and mainstream, White-Majority conditions. The second contrast tested a secondary hypothesis that perceptions of racism and anti-racism policy support will be greater among participants in the White-Majority condition than the control conditions. Although contrast analyses provide the primary tests of hypotheses, we follow convention and report results of omnibus ANOVA tests.

### Effects of the display manipulation

#### Perceptions of racism

The omnibus ANOVA revealed a significant effect of historical representation on racism perception, *F*_(2, 120)_ = 4.47, *p* = 0.013, $\eta_{\text{p}}^{2}$ = 0.069. The first contrast revealed the hypothesized effect of historical representation, *t*_(120)_ = 2.52, *p* = 0.013, *d* = 0.46. Participants exposed to Black-Majority representations of Black History perceived greater racism in U.S. society (*M* = 4.13, *SD* = 1.11) than did participants exposed to White-Majority BHM representations (*M* = 3.84, *SD* = 1.11) and participants in the control condition (*M* = 3.38, *SD* = 1.21). The second contrast indicated that the difference between the latter two conditions was also significant, *t*_(120)_ = 1.90, *p* = 0.060, *d* = 0.40. The pattern of means—greatest in the Black-Majority condition, followed by the White-Majority condition, and least in the control condition—suggests a linear trend in effectiveness of Black History displays in promoting perception of racism.

#### Support for anti-racism policies

The omnibus ANOVA revealed a marginal effect of historical representation on support for anti-racism policy, *F*_(2, 120)_ = 2.64, *p* = 0.075, $\eta_{\text{p}}^{2}$ = 0.04. The first contrast revealed the hypothesized effect of historical representation, *t*_(120)_ = 2.10, *p* = 0.038, *d* = 0.37. Participants exposed to Black-Majority representations of BHM indicated greater support for anti-racism policy (*M* = 4.01, *SD* = 1.21) than did participants exposed to mainstream, White-Majority representations (*M* = 3.77, *SD* = 0.93) and participants in the control condition (*M* = 3.47, *SD* = 1.01). The second contrast indicated that the difference between the latter two conditions was not significant, *t*_(120)_ = 1.359, *p* = 0.177, *d* = 0.0.31. This pattern of means suggests Black-Majority representations have greater effectiveness in promoting support for anti-racism policy than White-Majority representations and the control.

### Mediation analyses

To test the hypothesis that the effect of BHM representations on perception of racism mediates an indirect effect of BHM representations on support for remedial social justice policy, we used a statistical procedure for mediation analysis with a multicategory independent variable (Hayes and Preacher, 2014). Specifically, we created two contrast-coded variables from the three-level independent variable in the manner that we did for ANOVAs. This permitted a consideration of direct and indirect effects of Black-Majority BHM representations relative to the other two (i.e., White-Majority BHM and control) conditions.

**Figure 8:**
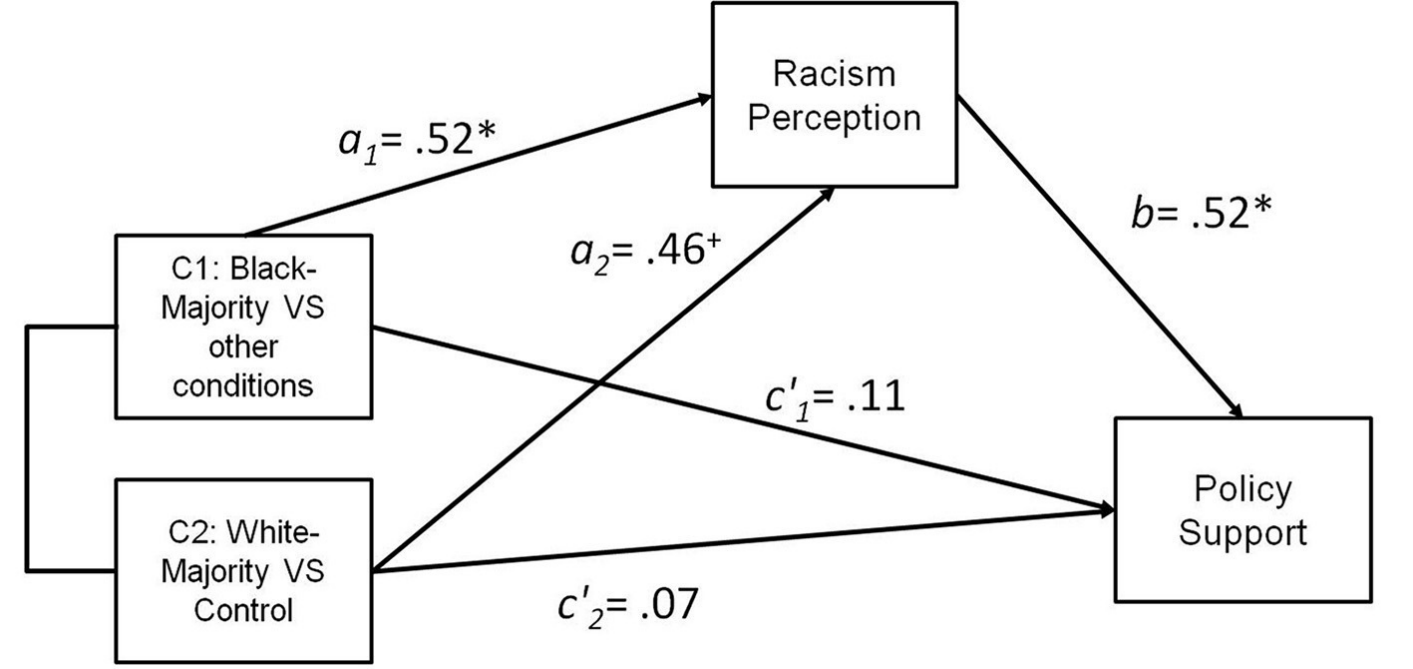
**Multicategorical mediation model of the effect of Black History Month displays on policy support via racism perception, Study 3**. Indirect effect 95% Confidence Interval [0.0490; 0.5177]. C1, contrast 1; C2, contrast 2; ^*^p < 0.05, ^+^*p* = 0.06.

Consistent with the contrast analysis, participants in the Black-Majority BHM condition perceived more racism [*b* = 0.53, *SE* = 0.10*, t*_(120)_ = 2.28, *p* = 0.024] compared to other conditions. In turn, racism perception significantly predicted support for anti-racist policy, [*b* = 0.52, *SE* = 0.07, *t*_(120)_ = 7.51, *p* < 0.001]. When accounting for the contrasts (independent predictors) and racism perception (proposed mediator) as simultaneous predictors of anti-racism policy, the overall model was significant, *R*^2^ = 0.35, *F*_(3, 119)_ = 21.39, *p* < 0.001. A bootstrapping analysis with 5000 iterations indicated that the relative indirect effect of the Black-Majority BHM representations (Contrast 1) through racism perception on anti-racism policy endorsement was significant (i.e., the 95% bias corrected confidence interval of 0.0490 to 0.5177 excludes zero), Figure 8. The relative direct effect of Black-Majority BHM representations was non-significant, *b* = 0.11, *SE* = 0.18, *t*_(120)_ < 1. This pattern of results is consistent with the interpretation that Black-Majority BHM representations promote support for anti-racism policy because they afford perception of racism.

### Open-ended responses

To explore the “active ingredients” of displays that produce differences in liking and recognition (in Study 2) or promote perception of racism and support for anti-racist policy (Study 3), we asked participants to indicate their favorite display and to describe what they liked about it. To analyze responses to this open-ended item, we developed a coding scheme based on *a priori* research questions and field observations from Study 1. Specifically, we created two categories to capture dynamics of critical, self-knowledge motivations vs. celebratory, self-enhancement motivations in BHM representations (e.g., Dagbovie, 2004; Journell, 2008; Chapman-Hilliard and Adams-Bass, 2015). Two independent raters used this scheme to code two thirds of the essays each: Rater A coded essays 1-45, Rater B coded essays 90-135, and both raters coded essays 45–90. Table 2 describes the binary coding categories (i.e., for judgments of absence/presence) and reports interrater reliability for each category based on the jointly coded one-third of essays. Discrepancies were resolved by averaging the coders' responses. We then summed across instances of the coding categories *struggle and historical barriers, historical racism, specific historical events, specific historical people*, and *educational value* to provide a total score for each participant on the dimension of critical self-knowledge. We summed across instances of *cultural diversity, inclusion/exclusion, aesthetic appeal*, and *neutral design* to provide a total score for each participant on the dimension of celebratory self-enhancement. The identity-relevance hypothesis suggests that celebratory self-enhancement themes will be more evident, but critical self-knowledge themes less evident, in responses to White-Majority displays than responses to Black-Majority displays.

**Table 2:** **Descriptions of essay coding categories and inter-rater reliability statistics, Study 3**.

**Category**	**Theme**	**Item scale**	**Kappa**
Mentions educational value or informativeness of display	CSK	0, no; 1, yes	0.526, *p* < 0.001
Mentions specific historical events or time periods	CSK	0, no; 1, yes	0.291, *p* = 0.037
Mentions specific and individual historical or contemporary people	CSK	0, no; 1, yes	0.928, *p* < 0.001
Mentions an instance of historical racism, discrimination, civil rights	CSK	0, no; 1, yes	0.000, *p* = 0.500^*^
Mentions themes of struggle, hardship, challenge or historical barriers African Americans face	CSK	0, no; 1, yes	0.023, *p* = 0.877^*^
Mentions aesthetic values or dimensions	CSE	0, no; 1, yes	0.615, *p* < 0.001
Mentions diversity values	CSE	0, no; 1, yes	0.459, *p* = 0.001
Mentions inclusion	CSE	0, no; 1, yes	0.692, *p* < 0.001
Mentions seemingly neutral, organizational, and structural aspects of the display	CSE	0, no; 1, yes	0.259, *p* = 0.082

*CSK, Critical Self Knowledge. CSE, Celebratory Self-Enhancement*.

* *Of the 44 overlapping essays, the coders agreed that 42 of the essays did not mention historical racism or historical barriers. The Kappas reflect a divergence on 2 essays. For racism, coder A indicated that 2 essays reflected this category while coder B did not. For barriers, coder A indicated that 1 essay contained this theme and coder B indicated that a different essay reflected this category*.

#### Critical self-knowledge

We operationalized critical themes as those that included consciousness of historical barriers and promoted a focus on Black History information and facts (e.g., specific events and individuals). Some participant responses reflected a focus on specific BHM content.

…there seems to be a lot of information in the center of the display. I liked that there are many different pictures of Rosa Parks and various magazine covers, newspapers and books, which shows what an important part she had in ending segregation [#15, White, female].

Similarly, the response of a participant in the Mainstream display condition reflected a focus on historical barriers faced by the Tuskegee Airmen.

“Remembering the Past …Shaping the future,” this quote next to the picture is important because we acknowledge what past struggles that have been made for African-Americans to give us the strength to continue to do better as a people [#107, Black, female].

#### Celebratory self-enhancement

We operationalized celebratory representations of BHM as those that deemphasized content in favor of broader inclusion, feel-good rhetoric (Trawalter et al., 2015), and a focus on relatively superficial aesthetic interests. In some cases, participants specifically commended a focus on broader diversity themes because they avoided negative information and afforded good feelings.

I liked the diversity theme. I feel that educating about others' cultures is best served on a more passive level rather than displays with the in your face approach. This display appeals to several minority groups, religious backgrounds, and achievements [#69, White, male].
I enjoyed the fact it did not push the thought of discrimination as much. The books shown show that it leaves the reader to find out about diversity and the African American movement. The display shows a door that can be opened to introduce African American information [#21, White, male].

#### Quantitative analyses of themes

To investigate whether responses to displays from different communities differed in the presence of these themes, we conducted a 2 × 2 mixed-model ANOVA with coding category (critical and celebratory) as the within-participant factor and historical representation condition (White-Majority and Black-Majority) as the between-participants factor. There was no main effect of condition, *F*_(1, 119)_ < 1.0, *p* = 0.60, but there was a main effect of coding category, *F*_(1, 119)_ = 10.57, *p* = 0.001, η_*p*_^2^ = 0.08. Generally, participants' open-ended responses referenced more celebratory themes (*M* = 1.60, *SD* = 0.90) than they did critical themes (*M* = 1.10, *SD* = 1.03), *t*_(120)_ = 3.86, *p* < 0.001, *d* = 0.36. The interaction was also significant, *F*_(1, 119)_ = 10.57, *p* = 0.001, $\eta_{\text{p}}^{2}$ = 0.08. Analyses to probe the interaction revealed support for hypothesized differences in display content. BHM displays from White-Majority schools (*M* = 1.79, *SD* = 0.87) elicited more celebratory themes in participants' open-ended responses than did displays from Black-Majority schools (*M* = 1.31, *SD* = 0.87), *t*_(119)_ = 2.94, *p* = 0.004, *d* = 0.55. In contrast, responses to displays from Black-Majority schools (*M* = 1.31, *SD* = 1.17) tended to include more critical themes than did responses to displays from White-Majority schools (*M* = 0.97, *SD* = 0.92), although this difference did not reach conventional levels of statistical significance, *t*_(119)_ = −1.82, *p* = 0.071, *d* = 0.33. These differences in frequency across open-ended responses provide some evidence that displays from different school settings bear (or afforded perception of) systematically different themes.

We propose that this difference between critical and celebratory representations of history might be the “active ingredient” that affords the differential effects of the displays that we observed in Studies 2 and 3.That is, displays from Black-Majority schools may have produced more dislike among White American students in Study 2 in part because they include more critical representations (that focus on historical barriers and racism) than do displays from White-Majority schools. Likewise, the relative emphasis on past racism in displays from Black-Majority schools may have alerted students in Study 3 to both the ongoing legacy of racism in the present and the need for energetic measures to overcome that legacy.

### Summary

Consistent with the cultural constitution hypothesis, results of Study 2 provide some evidence that BHM displays from Black-Majority schools were more effective at promoting perception of racism and support for anti-racist policy than were displays from White-Majority schools and a no-display control condition. Results also indicate that displays from White-Majority schools were somewhat effective at producing perceptions of racism relative to the no-display control condition, but just not as effective as displays from Black-Majority schools. As existing cultural products, representations of BHM from White-Majority schools may have included a mix of content, some of which promoted perception of racism and support for anti-racist policy and some of which promoted denial of racism and opposition to anti-racist policy. Indeed, analysis of open-ended responses reveal that critical history themes were present in White-Majority displays, although not to the same extent as in Black-Majority displays.

## Study 4

A primary strength of the preceding studies is ecological validity and the use of existing cultural products to examine the hypothesis that representations of history reflect and promote goals of different communities. Consistent with the psychological constitution side of the intentional worlds framework, Study 2 provides evidence that these cultural products bear the beliefs, desires, and preferences of people in the communities from which they originate. Consistent with the cultural constitution side of the intentional worlds framework, Study 3 provides evidence that these cultural products differentially promote identity-relevant action. A corresponding weakness of this “existing-products” approach is that, because displays from different schools incorporate many overlapping themes and elements, it is difficult to isolate the conceptually important features that serve as active ingredients that produce observed effects^5^. In order to provide a more precise test of the cultural constitution hypothesis and the role of different representations of history in promoting identity-relevant perception and action, Study 4 includes an experimental manipulation of celebratory and critical constructions of BHM.

## Method

### Participants

A total of 37 white American undergraduates (20 women and 17 men) from a predominately-White, Midwestern U.S. university ranged in age from 18 to 25 years of age (*M* = 19.54, *SD* = 1.52).

### Procedure

After obtaining informed consent, we assigned participants at random to one of three conditions associated with different constructions of American history. After exposure to one of the different sets of historical information, each participant completed measures of racism perception, policy endorsement, and demographics (similar to Study 3).

### Materials

#### Historical fact manipulation

The control condition modeled a “standard” American history approach and presented participants with 12 facts from which Black Americans and other minority groups were largely absent (e.g., *Benjamin Franklin, one of the most distinguished scientific and literary Americans of his era, was the first American diplomat*). The *historical achievements* condition replaced 5 of the 12 facts from the control condition with information about celebratory achievements of Black Americans to model a BHM approach found primarily in White-Majority schools and to a lesser extent in Black-Majority schools (e.g., *As a mission specialist aboard the Shuttle Endeavour, Mae Jemison was the first African American woman to enter outer space*). The *historical barriers* condition replaced the same 5 facts with critical historical information about racial barriers in American history to model a BHM approach primarily found in Black-Majority schools (e.g., *Dred Scott, a slave, sued for his freedom in 1847. The Supreme Court ruled that he was property and could not sue in federal court*). To encourage engagement with the facts, participants rated each fact on dimensions of importance and familiarity. After reading the facts, participants completed a questionnaire.

#### Perceptions of racism

We added six items to the racism perception measure from Study 3 (10 items; α = 0.82; 1, *not at all due to racism*; 7, *certainly due to racism*).

#### Support for anti-racism policies

The same four items in Study 3 assessed endorsement of policies aimed to address racial inequalities in the U.S. (α = 0.65; 1, *Strongly disagree*; 7, *Strongly agree*).

## Results and discussion

Again, we conducted ANOVAs with orthogonal planned contrasts using codes of (−1/3, −1/3, 2/3,) and (−1/2, 1/2, 0) for control, celebratory historical achievement, and critical historical barrier conditions, respectively. The first contrast tested the primary hypothesis that the relatively critical, barriers condition produced greater perceptions of racism and endorsement of anti-racism policy than the relatively sanitized, celebratory achievements and control conditions. The second contrast tested whether perceptions of racism and policy support differed for participants in the latter two conditions. Although these orthogonal planned contrasts provided the primary test of hypotheses, we again follow convention and report results of the omnibus ANOVA tests.

### Perceptions of racism

The omnibus ANOVA revealed a marginal effect of historical representation on racism perception, *F*_(2, 36)_ = 2.57, *p* = 0.092, $\eta_{\text{p}}^{2}$ = 0.13. A more precise test of the hypothesis comes from planned contrasts. The first contrast indicated that participants in the historical barriers condition (*M* = 4.74, *SD* = 0.79) perceived more racism than did participants in the historical achievements (*M* = 4.01, *SD* = 1.26) and control conditions (*M* = 3.91, *SD* = 0.80), *t*_(34)_ = 2.44, *p* = 0.02, *d* = 0.80. The second contrast was not significant, *t*_(34)_ < 1.

### Support for anti-racism policies

The omnibus ANOVA revealed an effect of historical representation on anti-racism policy support, *F*_(2, 34)_ = 3.66, *p* = 0.036, $\eta_{\text{p}}^{2}$ = 0.18 The first contrast revealed the hypothesized effect such that endorsement of anti-racism policies was greater among participants in the historical barriers condition (*M* = 4.29, *SD* = 0.84) than in the historical achievements (*M* = 3.21, *SD* = 1.19) and control (*M* = 3.54, *SD* = 0.97) conditions, *t*_(34)_ = 2.72, *p* = 0.010, *d* = 0.91. The second contrast was not significant, *t*_(34)_ < 1.

### Mediation analyses

We used the same procedure for mediation analysis with a multicategory independent variable (Hayes and Preacher, 2014) to test the hypothesis that the effect of information about historical barriers on perception of racism mediates an indirect effect of information about historical barriers on support for anti-racism policies. Specifically, we created two contrast-coded variables from the three-level independent variable to permit evaluation of direct and indirect effects of the historical barriers condition relative to the other two (i.e., historical achievements and control) conditions.

**Figure 9:**
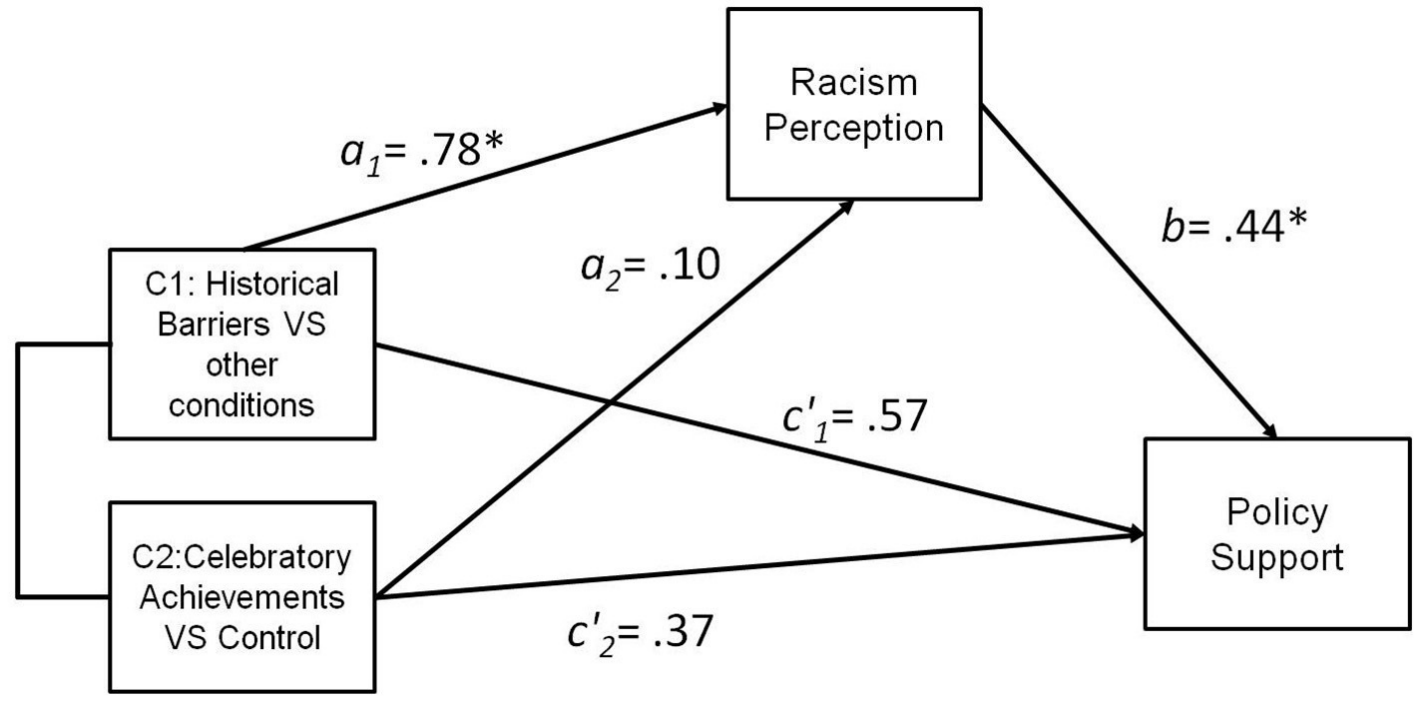
**Multicategorical mediation model of the effect of Black History Month facts on policy support via racism perception, Study 4**. Indirect effect 95% Confidence Interval [0.0307; 0.9485]. C1, contrast 1; C2, contrast 2; ^*^p < 0.05.

Consistent with the contrast analysis, exposure to information about historical barriers exerted a positive effect (compared to other conditions) on perception of racism [*b* = 0.78, *SE* = 0.35*, t*_(34)_ = 2.26, *p* = 0.031]. In addition, racism perception significantly predicted support for anti-racist policy, [*b* = 0.44, *SE* = 0.16, *t*_(34)_ = 2.69, *p* = 0.011]. When accounting for the contrasts (independent variables) and racism perception (proposed mediator) as simultaneous predictors of anti-racism policy, the overall model was significant, *R*^2^ = 0.34, *F*_(3, 33)_ = 5.30, *p* = 0.004. Bootstrapping tests with 5000 iterations indicated that the relative indirect effect of the critical historical barrier representations (Contrast 1) through racism perception on anti-racism policy endorsement was significant (i.e., the 95% confidence interval of 0.0307 to 0.9485 excludes zero), Figure 9. The relative direct effect of critical historical barrier representations non-significant, *b* = 0.57, *SE* = 0.35, *t*_(34)_ = 1.63, *p* = 0.11. This pattern of results is consistent with the interpretation that critical BHM representations depicting historical barriers increase support for anti-racism policy because they increase perceptions of racism.

### Summary

Similar to Study 3, participants in the historical barriers condition (modeled after BHM displays from Black-Majority schools) perceived more racism in ambiguous events and endorsed anti-racism policy to a greater extent than did participants in both the historical achievements (modeled after BHM displays from White-Majority schools) and control conditions. Perhaps reflecting more precise control over the content of representations, these effects were stronger in Study 4 than Study 3.

## General discussion

This research draws upon a diverse methodological toolkit—including qualitative field research, quantitative analyses, and experimental design—to investigate sociocultural variation in BHM representations and their consequences for perceptions of racial inequality in the U.S. The intellectual foundation of the project lies in the theoretical perspective of cultural psychology and its emphasis on the intentional character of everyday worlds. In one direction, (the psychological constitution hypothesis), the idea of intentional worlds emphasizes the *directed* character of everyday cultural ecologies. The relative prominence of different BHM representations in a given environment does not emerge by accident. Instead, the prominence or absence of particular ideas in material reality is the residue of purposeful activity: that is, the identity-consistent deposit of what seems good and true into local context. In the other direction (the cultural constitution hypothesis), the idea of intentional worlds emphasizes the *directive* character of everyday cultural ecologies. The BHM representations prominent in different ecologies are not the inert end-product of previous activity. Instead, they systematically afford courses of action consistent with identity concerns of different communities.

Support for the psychological constitution hypothesis came from field research in public schools (Study 1) and participants' open-ended reactions to photographs (Study 3). Celebratory BHM representations were most commonly found in material from White-Majority schools, and BHM representations highlighting historical racism were most commonly found in Black-Majority schools. Study 2 provides additional support for the psychological constitution hypothesis. White American participants expressed greater positive affect, familiarity, and liking for White-Majority BHM displays than Black-Majority BHM displays. Moreover, the relationship between White American identification and responses to BHM representations was significantly more negative in response to displays from Black-Majority schools than in response to displays from White-Majority schools. Together, these results suggest the extent to which BHM displays are not identity-neutral. Instead, displays from White-Majority settings reflect and objectify preferences and understandings that resonate with dominant constructions of White Americans identity. Displays from Black-Majority settings reflect and objectify preferences and understandings that conflict with dominant constructions of White American identity.

Studies 3 and 4 provide support for the cultural constitution hypothesis. In Study 3 we exposed participants to existing BHM displays from different communities to investigate the extent to which these cultural tools afford different tendencies of racism perception and policy support. Results indicated that exposure to the BHM displays from Black-Majority schools (i.e., the same representations for which White Americans expressed dislike in Study 2) led subsequent participants both to perceive a greater role of racism in American society and to express greater support for anti-racism policy than exposure to both BHM displays from White-Majority schools (i.e., the same ones for which White Americans expressed a preference in Study 2) and a no-display control condition. In Study 4, we experimentally manipulated exposure to themes that we distilled from existing representations. Results again indicated that exposure to critical history representations that emphasized historical barriers led subsequent participants to perceive greater influence of racism in American society and to express greater support for anti-racism policy than did exposure to both celebratory BHM representations that emphasized individual achievements (i.e., a theme that was more typical of displays from Black-Majority schools than White-Majority schools) and representations that made no mention of Black contributions to American history.

Together, these results suggest the extent to which BHM displays are not psychologically inert objects. Instead, displays from different communities bear beliefs and desires of their producers that systematically direct perception and action toward different ends. White Americans preferences (in Study 2) for BHM displays from majority White American schools are not accidental. Instead, whether or not they are conscious of the source of their preferences, White American undergraduates may prefer these displays precisely because these displays afford denial of racism, opposition to anti-racism policy, and preservation of the system of racial domination from which they benefit. As students and teachers act on these preferences, choose to emphasize diversity or individual accomplishments, and omit information about racist barriers, they selectively reproduce cultural worlds of sanitized history representations that in turn afford denial of racism and weak support for anti-racism policy.

### Limitations and future directions

Although results are consistent with hypotheses, the research is not without limitations. We mention three that constitute important directions for future research.

One limitation is the basis in a relatively narrow sample of BHM displays from schools in a single U.S. metropolitan area. Accordingly, we make no claims that results reflect the broader distribution of BHM themes across a wider set of cultural products and cultural ecologies. Indeed, we are doubtful of any claims about characteristic tendencies that apply uniformly across African American or European American settings. Variation in BHM constructions within and across communities (and over time) remains a fruitful direction for research.

Another limitation concerns the narrow sample of participants (e.g., along dimensions of race-ethnicity, age, and educational background). Although the focus on White American spaces is appropriate for addressing our theoretical interest in historical representations as tools of domination, an important task for future research is to consider identity-relevant influences on reproduction of historical representations among people from historically oppressed groups. For example, an interesting question for future research is the extent to which dynamics of BHM commemoration among Black Americans vary as a function of the “double consciousness” (DuBois, 1989/1903, p. 5) associated with American-ness and Black-ness. Black American students vary in preferences for Black History as a function of their prior racial socialization experiences (Thornhill, 2014), and their preferences may change across the life course if they move from predominantly Black American to predominantly White American spaces (Starck et al., unpublished manuscript).

Another reason to consider a broader diversity of participants concerns consequences of engagement with different BHM constructions. The present research demonstrates that an emphasis on critical history narratives promotes greater awareness of racism in contemporary U.S. society. Although we have interpreted this outcome in a relatively positive light (as a precursor to anti-racist action among White Americans), previous research has emphasized that racism perception can put people with marginalized identities at risk for negative psychological outcomes (e.g., Pinel, 1999; Mendoza-Denton et al., 2002; cf. Tatum, 1997). Extension of the present research to participants with marginalized identities would permit investigation of this important dilemma that parents and educators face regarding socialization about racism (Tatum, 1987; Hughes et al., 2006).

Finally, our investigation focused on the implications of historical representation for perception of racism and support for anti-racist policy. We did not consider racist attitudes or other individual difference measures that have figured more prominently in social psychological research. Our focus on outcomes of racism perception and support for anti-racist policy is consistent with emerging perspectives that express misgivings about constructions of racism as a matter of individual prejudice (i.e., “prejudice problematic”; Wetherell and Potter, 1992; see also Leach, 2002; Paluck, 2009; Wright and Lubensky, 2009; Hammack, 2011; Dixon and Levine, 2012). However, it remains unclear whether the patterns we observed in the present research would extend to measures of individual prejudice.

## Conclusion: the intentionality of everyday worlds

Without downplaying limitations, the primary contribution of the present project is to provide a dynamic account of the mutual constitution of culture and psyche at the “ecological” level of local affordances and everyday activity. In the process, the project illuminates a cultural psychological approach to the collective character of psychological experience.

### Cultural dynamics

Social-psychological research in the field of cultural psychology has typically emphasized comparison across settings of psychological tendencies (and occasionally, cultural products; Snibbe and Markus, 2005; Markus et al., 2006; Miyamoto et al., 2006; see Morling and Lamoreaux, 2008). Such work has been indispensable for illuminating both sociocultural variation in psychological processes and the particular constructions of reality that underlie conventional scientific wisdom. Critics of this work have noted that it often presents an overly static and essentialist understanding of culture associated with Orientalism and other kinds of cultural imperialism (e.g., Gjerde, 2004; Okazaki et al., 2008). Against this background, the present work contributes to a more dynamic account of cultural variation that emphasizes the active, mutual constitution of cultural context, and psychological experience (Shweder, 1990; Markus and Hamedani, 2007).

Regarding the cultural constitution hypothesis, our investigation focuses less on broad, national spaces, and instead emphasizes a more local form of cultural-ecological variation: the cultural practices and material products of BHM displays in schools serving different communities. This emphasis resonates with ecological perspectives that consider the mutual constitution of organism and habitat at a more “micro” level of analysis than is typical in canonical forms of cultural psychology (Barker, 1968; Gibson, 1977; with respect to cultural psychology, see also Uskul et al., 2008; Oishi and Graham, 2010). It likewise makes contact with sociocultural-historical perspectives in cultural psychology (e.g., Cole, 1995) that emphasize cultural constitution of psychological experience via mediation of cultural tools. The result is a dynamic account of cultural psychology that avoids broad, essentialist generalizations about reified, monolithic groups.

Regarding the psychological constitution hypothesis, our investigation focuses on the active reproduction of everyday worlds through repeated acts of preferential selection. Although previous work has emphasized the role of social cognitive processes or personal motivations on preferential selection of cultural units (e.g., Zajonc, 1980; McIntyre et al., 2004; Savani et al., 2008), the present project extends consideration to the role of social identification and associated motivations in preferential reproduction of cultural reality (e.g., “To what extent would you like to see this display in your former high school”). By considering ways in which people who occupy positions of dominance act on identity considerations to reproduce identity-charged realities, the present research lays the foundation for a cultural psychology of power, privilege, and oppression. More generally, this focus on preferential selection and active maintenance contributes to a dynamic account of cultural psychology that retains an appreciation for human agency (Gjerde, 2004).

### Collective self-regulation

An important implication of this account is that even when everyday realities appear to be static and unchanging across generations, this apparent stability masks dynamic activity as people repeatedly select (or de-select) features that resonate with their understandings and desires. Moreover, the results of these acts of preferential selection (or de-selection) are not merely end-products of a behavioral cycle; instead, they construct everyday realities that constitute the “facilitating conditions” (Kroeber and Kluckhohn, 1952) for future action. With respect to the present work, identity-relevant acts of preferential selection produce everyday worlds that are *intentional* in two important senses. First, these worlds are “intentional” because they are *directed*; that is, people in the course of everyday life constantly re-fashion worlds that bear their identity-enhancing beliefs and desires. Second, these worlds are “intentional” because they are *directive*; that is, they nudge subsequent action toward identity-enhancing ends. The intentionality of these behavioral products is an underappreciated feature of psychological experience with important implications for theorizing collective manifestations of mind.

Consider the topic of memory. The bidirectional relationship between memory and identity has been an enduring theme of psychological research. Yet, most discussions have considered how individuals reconstruct autobiographical memories in ways that serve present identity concerns or how different autobiographical memories have implications for experience of personal identity (Wilson and Ross, 2003). Recent work has begun to explore this relationship at the level of collective self, investigating how different understandings of history both reflect and impact present experience of social identity (Wertsch, 2002; Liu and Hilton, 2005; Roccas et al., 2006; Sahdra and Ross, 2007; Blatz and Ross, 2009; Licata and Klein, 2010; Figueiredo et al., 2011; Gunn and Wilson, 2011). In many of these discussions, *collective* refers to individual experience of identity or memory when people imagine themselves in terms of a social identity category.

A cultural psychology emphasis on the intentionality of everyday worlds provides a broader understanding of *collective* as a process that is evident not only when people imagine themselves in terms of a social identity category, but any time that they appropriate cultural tools and other culturally evolved affordances (Rogoff, 2003). Thus, the collective character of memory is not merely evident when categorizing self in terms of social identities, but more generally any time a person appropriates psychologically constituted, cultural affordances for understanding the past. This distinction becomes especially important for understanding the concept of *collective self-regulation* in referencing the present work. We propose that the present work is an investigation of collective self-regulation not in the sense of regulation of the collective self, but instead in the double sense of collective regulation of collective self.

To illustrate, consider a teacher who commemorates BHM with mass-marketed, mainstream artifacts that—by focusing on individual achievements of black heroes without mentioning the racism that required heroic resistance—reflect and promote racism denial. Even if the teacher displays these artifacts without personal motivations to deny the extent of racism, the products she deposits nevertheless bear identity-defensive beliefs and desires of the people who produced them (see Study 2). Similarly, even if the teacher personally intends to promote awareness of racism and support for reparative policy, her actions nevertheless reconstitute ecologies filled with cultural tools that promote denial of racism and opposition to reparative policy (as in Studies 3 and 4). In other words, the motivations and intentions associated with the teacher's action are not reducible to her personal motivation to deny racism or personal intention to oppose antiracist policy; instead, the relevant motivations and intentions reside in the cultural tools for memory and identity on which the teacher draws.

We refer to this process as collective self-regulation not only because the memory, motivation, and emotion refer to “collective self,” but also because the ways in which people control and direct actions of others is both (a) collaborative or distributed across multiple actors and (b) embedded within the cultural tools. The directive force of these everyday affordances need not work through the individual intentions of an individual actor; instead, regardless of one's personal motivation or intention, engagement with material traces of psychologically constituted, cultural affordances direct one's action toward identity-relevant ends. As such, the present work highlights a process of collective self-regulation that operates not as individual regulation of the collectively-identified self, but instead as intentional worlds that are effective regulatory tools regardless of whether any given individual shares the motivations, intentions, or even identification patterns of the original actors.

This idea of collective self-regulation is central to both Woodson's vision for the cultural practice of BHM and mainstream appropriation of this practice. Social justice advocates propose that “recovering historical memory” (p. 31, Martín-Baró, 1994; see also Chapman-Hilliard and Adams-Bass, 2015) is essential for liberation and community healing. BHM commemorations that recognize (rather than repress) collective memories of historical injustice are cultural tools that alert people to occurrences of present injustice, provide alternative (less victim-blaming) explanations for present inequalities, motivate the desire for social change, and otherwise regulate action toward social justice ends. At the same time, the present work cautions that not all representations of BHM will contribute equally to this goal. Indeed, selective pressures in mainstream society shape reactionary development of BHM representations that serve as tools for maintenance and reproduction of racial domination.

## Author contributions

This manuscript is based on a Ph.D. dissertation that PS submitted to the University of Kansas. PS conducted data collection and wrote the initial draft. PS and GA both contributed to study design, data analysis, and revision of subsequent drafts.

### Conflict of interest statement

The authors declare that the research was conducted in the absence of any commercial or financial relationships that could be construed as a potential conflict of interest.

## Footnotes

^1^Throughout this manuscript, we refer to Black History Month displays from schools in which Black students are in the numerical majority as “Black-Majority” schools. This includes one school in which Hispanic students were in the majority, followed by Black students, and then by White students.

^2^For ease of communication, we focus here on the CSE *identity* subscale for White racial/ethnic belonging. However, we also included the *private collective self-esteem* subscale and a version of these CSE subscales that assessed connection to American national identity. Patterns for these subscales are similar to those that we report here. Details of analyses involving these additional dimensions of identification are available from the authors.

^3^The demographic questionnaire included questions about the racial/ethnic background of participants' educational experience in studies 2 and 3. We asked what % of the students at different schooling levels were White (e.g., high school; Sanders-Thompson, 1995). Supplementary analyses of these data are available from the authors. Results indicated that reported racial/ethnic background of high school was not correlated with the primary dependent variables in Study 2 (affective responses, display evaluations) or Study 3 (racism perceptions, policy support). This question was not included in Study 4.

^4^Five of these displays are materials that the first author obtained in Study 1 and that participants rated in Study 2. We added the sixth display (and did not use one of the seven displays from White-Majority schools in the White-Majority condition) in order to equate procedures across conditions. This sixth display came from a teacher at a predominately Black school in the same metropolitan area who sent pictures of the school's BHM display after we completed Study 1.

^5^As an example of the ways in which different artifacts can contain mixed messages, consider again the psychological consequences of the celebratory, achievement-oriented poster that Target department stores created and distributed for BHM (Figure 1). Presumably, the purpose of this representation is to highlight Black American achievements and thereby promote a sense of pride and collective self-efficacy. However, the poster also re-presents a sanitized version of American history that fails to emphasize racist barriers and anti-racist struggle. Moreover, the tagline—“They didn't wait for opportunity. They invented it”—carries the individualist implication that true heroes do not complain about structural barriers or rely on collective solutions; they overcome by force of individual will. Such celebratory, sanitizing narratives maintain the status quo and reproduce racial inequality by promoting strategies of individual mobility within the system at the expense of collective action to change the system (Pitre and Ray, 2002; Schick and St. Denis, 2005; Tajfel and Turner, 1986).
